# Calcium signaling from sarcoplasmic reticulum and mitochondria contact sites in acute myocardial infarction

**DOI:** 10.1186/s12967-024-05240-5

**Published:** 2024-06-09

**Authors:** Esther Densu Agyapong, Gaia Pedriali, Daniela Ramaccini, Esmaa Bouhamida, Elena Tremoli, Carlotta Giorgi, Paolo Pinton, Giampaolo Morciano

**Affiliations:** 1 Department of Medical Sciences, University of Ferrara, Ferrara, Italy; 2 Maria Cecilia Hospital, GVM Care&Research, Cotignola, Italy

## Abstract

Acute myocardial infarction (AMI) is a serious condition that occurs when part of the heart is subjected to ischemia episodes, following partial or complete occlusion of the epicardial coronary arteries. The resulting damage to heart muscle cells have a significant impact on patient’s health and quality of life. About that, recent research focused on the role of the sarcoplasmic reticulum (SR) and mitochondria in the physiopathology of AMI. Moreover, SR and mitochondria get in touch each other through multiple membrane contact sites giving rise to the subcellular region called mitochondria-associated membranes (MAMs). MAMs are essential for, but not limited to, bioenergetics and cell fate. Disruption of the architecture of these regions occurs during AMI although it is still unclear the cause-consequence connection and a complete overview of the pathological changes; for sure this concurs to further damage to heart muscle. The calcium ion (Ca^2+^) plays a pivotal role in the pathophysiology of AMI and its dynamic signaling between the SR and mitochondria holds significant importance. In this review, we tried to summarize and update the knowledge about the roles of these organelles in AMI from a Ca^2+^ signaling point of view. Accordingly, we also reported some possible cardioprotective targets which are directly or indirectly related at limiting the dysfunctions caused by the deregulation of the Ca^2+^ signaling.

**Supplementary Information:**

The online version contains supplementary material available at 10.1186/s12967-024-05240-5.

## Introduction

Acute myocardial infarction (AMI) represents a consequence of ischemic heart diseases (IHD), mainly caused by the partial or the complete blockage of the epicardial coronary arteries leading to the death of myocardial cells. These cells are replaced by fibrotic tissue, resulting in a loss of cardiac contractility. AMI accounts for over 33% of mortality associated with IHD [1]. It can be classified into two types: ST-segment elevation myocardial infarction (STEMI) and non-STEMI (NSTEMI), based on the electrocardiographic presentation during diagnosis. The severity of ST-segment changes is influenced by the location of the myocardial region affected by acute ischemia [2, 3]. Patients with STEMI are at higher risk of mortality due to complete thrombotic occlusion of the vessel, transmural infarction, and the absence of collateral circulation [4]. On the other hand, patients with non-STEMI experience reduced blood flow in the affected coronary artery without complete occlusion, but they face higher long-term mortality risk due to the potential occurrence of multivessel coronary artery disease [5]. Throughout this review, we will focus mainly on STEMI cases because the total occlusion of the vessel greatly impacts on the biochemical pathways of interest, highlighting them. The ischemic episode is primarily caused by plaque rupture, leading to the formation of blood clots that reduce microcirculatory perfusion and limit blood flow to the heart. This imbalance between oxygen supply and demand accounts for about 70% of fatal events [6]. Then, reperfusion strategies, such as pharmacological approaches or percutaneous coronary intervention (PCI) and coronary artery bypass, have become the gold standard for treating AMI [6].

Early reperfusion has been demonstrated to limit ischemic cell death, but it can also result in cellular alterations and further tissue damage. The resulting damage can be categorized as reversible or irreversible. Reversible damage allows cardiac myocytes in the affected area to survive ischemia, and early reperfusion facilitates the recovery of cellular function. However, irreversible damage causes complete loss of resilience in myocytes, leading to cardiac tissue death. Irreversible damage is characterized by various changes in cardiomyocyte structure, including cell swelling, denaturation of intracellular proteins, and cell calcification [7]. During hypoxia, cardiomyocytes undergo anaerobic metabolism, leading to ATP production deficiencies, ion imbalances, and intracellular acidosis. Upon blood flow restoration, rapid reoxygenation of cardiac tissue restores ATP production and pH levels but also leads to the hyperproduction of reactive oxygen species (ROS) and intracellular calcium (Ca^2+^) overload, ultimately causing cardiomyocyte death and systolic dysfunction [8]. Arrhythmogenesis, whose onset is very common after ischemia and at reperfusion time, represents one of these dysfunctions. Here, mitochondria might have a central role through the control of the metabolism-excitation route, despite it is not yet fully clarified. Indeed, some mitochondrial-localized channels (i.e., inner membrane anion channel) when inhibited help in maintaining the mitochondrial membrane potential (Ψ_M_) and action potentials during stress. Moreover, also Ca^2+^ and (mitochondrial ROS) appear to be determinant, by promoting mitochondrial dysfunctions dependent from a vicious cycle of Ca^2+ ^overload and mitoROS production, consequent Ryanodine receptor 2 (RyR2) oxidation and Ca^2+ ^leak from the sarcoplasmic reticulum (SR) [9].

**Fig. 1:**
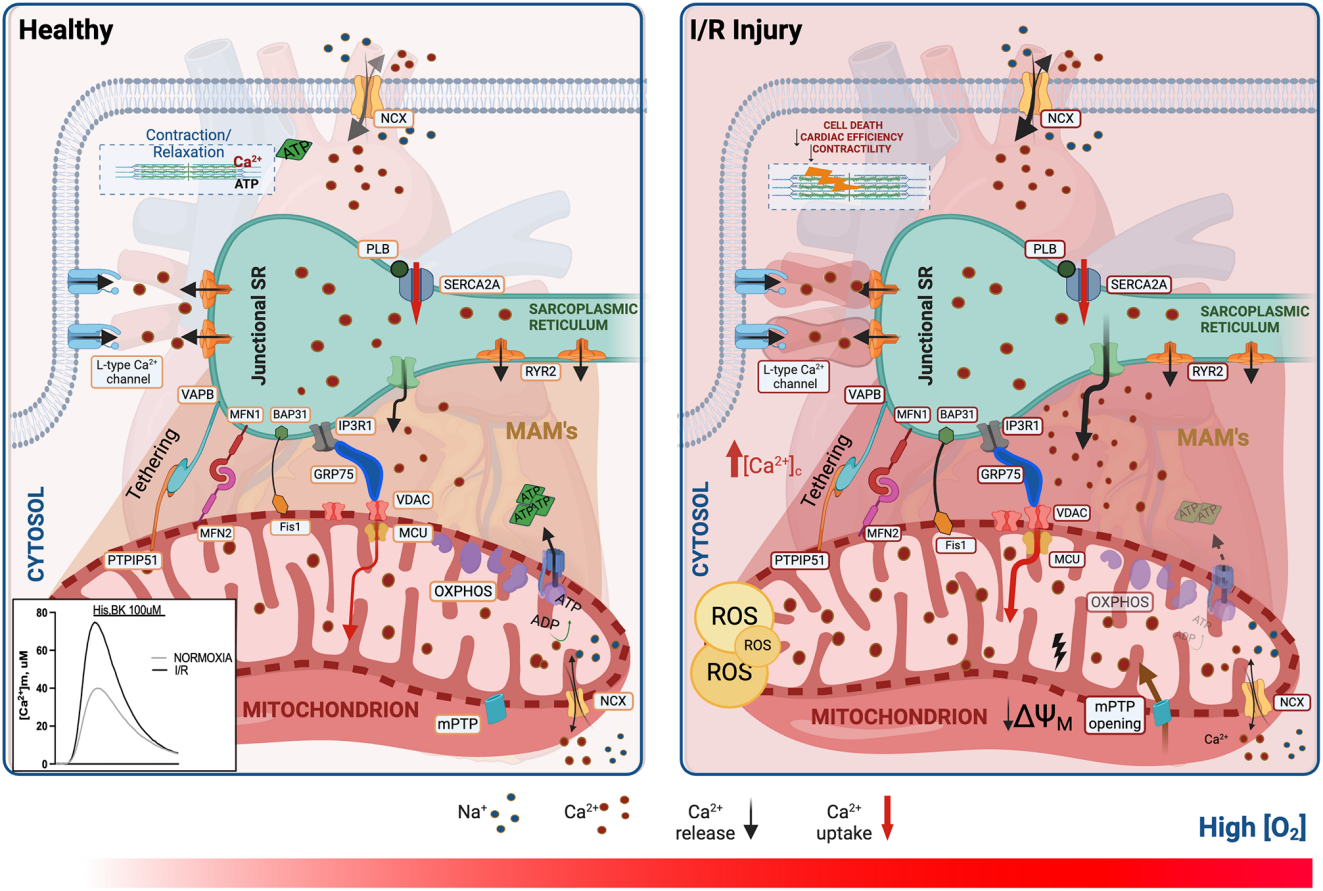
Representation of the Ca^2+ ^signaling between mitochondria and sarcoplasmic reticulum in healthy condition and after IRI This figure illustrates the dynamics of calcium (Ca^2+^) signaling between the sarcoplasmic reticulum (SR) and mitochondria associated membranes (MAMs) under normal physiological conditions and following ischemia-reperfusion injury (IRI). In healthy conditions, Ca^2+ ^ions are released from the SR via channels such as the inositol 1,4,5-triphosphate receptor type 3 (IP3R3), inositol 1,4,5-triphosphate receptor type 1 (IP3R1), and ryanodine receptors (RyR2). These Ca^2+ ^ions can then enter the mitochondria through the voltage-dependent anion-selective channel (VDAC) and the mitochondrial calcium uniporter (MCU). Following IRI, alterations in Ca^2+ ^handling occur, affecting the balance between the SR and mitochondria. This dysregulation may involve changes in the activity of the Na^+^/ Ca^2+ ^exchanger (NCX), plasma membrane Ca^2+ ^ATPase, and sarcoplasmic reticulum Ca^2+ ^ATPases (SERCA), as well as modulation of the phosphorylation status of phospholamban (PLB). Disruption of Ca^2+ ^homeostasis during IRI can lead to mitochondrial dysfunction such as excessive reactive oxygen species (ROS) production, mPTP opening, impairment of ATP production and cell death

As a messenger of cell death, but also stimulator of ATP synthesis and cardiac contraction [10], Ca^2+^ is sensed and captured by mitochondria from different intracellular stores (i.e., SR) and the extracellular milieu (Fig. 1). The events associated with ischemia reperfusion (I/R) have a strong impact on Ca^2+^ homeostasis, causing a variation in the expression and functionality of the proteins that bind and transport Ca^2+^; therefore, these changes in the intracellular Ca^2+^ fluxes can influence cell fate.

In this review, we will summarize the impairments in charge of mitochondria, SR and their contact sites named as mitochondria associated membranes (MAMs), that could be clinically relevant in cardiac I/R. We will highlight biochemical events regarding Ca^2+^ signaling dysregulation as contributor of cardiac damage, and whether they can be successfully targeted to reduce reperfusion injury. Despite advancements, patients with AMI continue to face substantial risks of mortality and morbidity. Novel treatments are imperative to shield the myocardium from the adverse impacts of AMI and reperfusion injury, aiming to minimize infarct size (IS), to sustain cardiac function, and to forestall heart failure onset [11, 12].

### The importance of sarcoplasmic reticulum and mitochondria for Ca^2+^ homeostasis in acute myocardial infarction

The balance of intracellular Ca^2+^ at any level is crucial for normal cardiomyocyte function, from embryos to adulthood [13]. One of the main functions of the sarcoplasmic reticulum (SR) is to finely regulate the accumulation and the release of this ion close to the subsarcolemmal space, playing a key role in excitation-contraction coupling (ECC) in the heart. The importance of Ca^2+^ handling at SR and T tubules (TT) interface in ECC is widely reviewed elsewhere and it is already a reliable molecular target for cardioprotective drugs. What is almost unknown to date is the contribution of the dysregulation of Ca^2+^ signaling at the SR-mitochondria interface in ECC. Indeed, whether this pattern is essential for cardiac excitation-metabolism coupling, still few evidence connects mitochondria Ca^2+^ buffering to heart contraction. A seminal paper by Rizzuto’s group, in 2012 provided the first pioneer proof that mitochondria are able to taken up a significant fraction of Ca^2+^ released during systole and then released back into the cytosol during diastole [14]; this happens through the mitochondrial calcium uniporter (MCU) complex, a channel of the inner mitochondrial membrane (IMM). Moreover, the MCU-mediated Ca^2+^ uptake into mitochondria is of crucial importance for the direct ATP synthesis [15] and for the activation of dehydrogenases that feed electrons into the respiratory chain. Of note, MCU is essential for the control of “fight or flight” mechanisms, by regulating the Ca^2+^-mediated heart rate increase in cardiac pacemaker cells during stress [16]. Further investigations encompassing these noteworthy findings have been poor in the pathological field perhaps leaving the real role of the mitochondrion in ECC unknown. As mitochondria receive Ca^2+^ from SR, cytosol and from microdomains that are formed at MAMs, essential is the evaluation of what changes in correspondence of this route during AMI.

The proximity between mitochondria and SR facilitates reciprocal exchange between organelles, enabling tight integration between ATP generation by mitochondria and Ca^2+^ release mediated by Ca^2+^ channels in SR membrane, meeting the energy demands of the myocyte [17]. The activity of various mitochondrial enzymes in oxidation is influenced by the concentration of Ca^2+^, suggesting that an increase in Ca^2+^ influx into mitochondria promotes electron transport and ATP production. Indeed, it is reported that tricarboxylic acid (TCA) cycle is modulated by Ca^2+ ^levels, in particular, the activation of ketoglutarate dehydrogenase (KGDH), isocitrate dehydrogenase (IDH) and pyruvate dehydrogenase (PDH) are directly dependent from matrix Ca^2+ ^concentration [18, 19]. This leads to an increase in NADH, higher activity of the ETC and a positive loop for what concern mitochondrial metabolism. An additional dehydrogenase, named as FAD-glycerol phosphate dehydrogenase (GPDH) and localized in the IMM, mediates the transfer of reducing equivalents from NADH to the ETC by increasing ATP production [20, 21].

Overall, evidence of the Ca^2+^-dependent modulation of the oxidative phosphorylation derived by studies on skeletal muscle, which described an augmentation of conductance of the complexes I, III and IV of ETC [22]. Furthermore, experiments on isolated porcine heart mitochondria demonstrated that Ca^2+ ^directly stimulates ATP synthase (complex V), enhancing respiratory chain activity and increasing ATP production [23].

However, intense ETC activity leads to increased production of mitoROS, which can overwhelm cellular antioxidant defenses, causing harmful damage, particularly during I/R when mitochondrial Ca^2+^ overload correlates with a significant increase of ROS and oxidative stress [24].

As anticipated above, RyR2 is a Ca^2+^ channel that can be found also in close proximity to MAMs to guarantee the correct release of Ca^2+^ towards mitochondria. Functional anomalies in RyR2 Ca^2+^ release have a noteworthy symptomatic impact in cardiac disease [9]; in a post-MI murine model, sustained Ca^2+ ^leakage through RyR2 from SR leads to mitochondrial Ca^2+^ overload and a consequent mitoROS production, which cause oxidation of the receptor and an aberrant Ca^2+^ leak, creating a feedback loop [25]. RyR2 contain multiple cysteine residues, making them susceptible to oxidative changes. Their sensitivity to redox alterations means that the effects of ROS on RyR2 can vary based on concentration. Low levels of oxidants can boost RyR2 activity, while persistent high levels lead to irreversible inhibition, possibly affecting individual cysteine residues differently. Various oxidative modifications like S-glutathionylation and S-nitrosylation impact RyR2 function [23]. Additionally, oxidative stress can activate RyR2 by promoting disulfide bond formation between subunits, altering the channel’s structure and reducing SR Ca^2+^. Modulation of mitochondrial Ca^2+^ levels could be regulated also by RyR2 because, upon activation, RyR2 releases Ca^2+^ ions into the cytoplasm, which can subsequently enter the mitochondria through MCU [24]. This suggests that modulation of RyR2 function may have a protective effect against mitochondrial Ca^2+^ overload that occurs during IRI. Further evidence showed that RyR2 oxidation increases channel activity and worsens defective intracellular Ca^2+^ homeostasis [25].

In a time course manner after AMI, RyR2 expression was monitored with the recording of a transient decrease, followed by an increase only during the 4-week recovery, suggesting a role of this channel in the compensatory reaction of rat cardiac tissue to injury [26]. Of note, during I/R RyR2 is reported to be phosphorylated by Ca^2+^/calmodulin-dependent protein kinase II (CaMKII) at S2814; this site of phosphorylation is crucial for the cardiac damage probably linked to the increase in SR Ca^2+^ leak and consequent mitochondrial Ca^2+^ overload. Knock-in mice carrying a genetically inactivated site on RyR2 revealed a strongly reduced necrosis and apoptosis, thus a protection from I/R injury (IRI). By contrary, knock-in mice with the constitutively activated form of the aminoacidic site, presented a much more severe phenotype [27]. Accordingly, several studies demonstrated that ablation of CaMKIIδ, the main isoform in the myocardium, protects against I/R, suggesting a promising target for heart protection [28, 29].

Aberrations of other SR Ca^2+^ handling proteins are associated with AMI, such as SERCA2a. This pump is essential in controlling systole and diastole events as it modulates the cytosolic Ca^2+^ and thus the amount of Ca^2+^ bound to Troponin C. During I/R, there is an impairment of the normal influx of Ca^2+^ resulting in an overload of the amount of intracellular Ca^2+^ and a decrease in the activity of the SERCA2a isoform [30, 31]. Several studies over the years have demonstrated that increased SERCA2a expression can improve myocardial contractility and Ca^2+^ management following I/R myocardial injury [32, 33]. Of note, SERCA2a affinity for Ca^2+^ is carefully modulated by the interaction with phospholamban (PLB), whose expression levels and the phosphorylation status may limit or allow Ca^2+^ reuptake into the SR. This mechanism is regulated either by protein kinase A (PKA) or by CaMK, both proteins can phosphorylate PLB, releasing its inhibitory effects on SERCA2a [34]. A study by Shintani-Ishida demonstrated a significant PLB dephosphorylation associated to ischemic episodes in an in vivo model of AMI, that allows to cytosolic Ca^2+^ overload in early reperfusion, contributing to the formation of contraction bands [35].

The contractile dysfunction encountered following I/R and widely described until now can be further ascribed to the proteolytic modification of SR proteins like Junctophilins (JPH) 1 and 2 [36], calcineurin, protein kinase C (PKC), SERCA2a. This happens following the activation of Ca^2+^-dependent proteases, such as calpains, during Ca^2+^ overload that occurs in I/R [37]. Indeed, both JPH1 and JPH2 proteins are recognized as target of calpain-1 and 2, whose activity enhances during stress conditions [38]. Their expression, in particularly of JPH2, is downregulated during IRI [38].

Of note, in I/R, mitochondria are the first sensing organelles of the lower presence of oxygen; the ETC activity is inhibited and a consequent significant depletion in ATP production occurs. In this condition the cell favorizes a metabolic switch to anaerobic glycolysis. This leads to an intracellular Na^+^ and Ca^2+^ overload as a compensatory mechanism to buffer the excessive H^+^, mediated respectively by the activation of the Na^+^/H^+^ exchanger [39], inhibition of Na^+^/ Ca^2+^ antiporter (NCX) [40, 41]. Intracellular Ca^2+ ^levels and membrane depolarization can also regulate the activity of large-conductance calcium-activated potassium ion channels (BKCa channels). These channels participate in a wide variety of fundamental physiological processes from vascular tone and cardiac rhythmicity [42]. BKCa channels have been detected in the IMM of adult cardiomyocytes where they increases K^+^conductance and improves mitochondria respiratory function by reducing the production of mitoROS and decreasing deleterious intra-mitochondrial Ca^2+ ^accumulation occurring after I/R injury [43–45]. Increased production of mitoROS especially during cardiac reperfusion triggers the opening of another channel: the mitochondrial permeability transition pore (mPTP) [40, 41]. Opening of the mPTP is characterized by depolarization of the Ψ_M_and from to exposure to high levels of mitoROS. During AMI, oxygen deprivation impairs oxidative phosphorylation, causing ion dysregulation. During reperfusion, the oxygen-starved heart undergoes oxidative damage due to the sudden abundance of nutrients and O_2_, triggering mPTP-dependent cell death [46, 47]. The intricate relationship between malfunctioning mitochondria and disrupted Ca^2+ ^regulation underscores the intricacy of cardiomyocyte death during myocardial reperfusion injury. Key contributors to cell death in this context include hypercontracture and mitochondrial permeability transition (MPT), both exacerbated by incomplete repolarization of mitochondrial membranes. Hypercontracture arises from excessive contractile activity promoted by restored energy production in presence of elevated cytosolic Ca^2+ ^levels. However, in tissues, the mechanical strain stemming from neighboring cell hypercontraction leads to mutual cellular breakdown and necrosis [48].

Whether the MCU-mediated Ca^2+^ influx is confirmed to be an important regulator of cardiac metabolism (i.e., ATP production) and in heart contractility in response to mechanisms of mild stress, its role in I/R remains controversial, at least from the analysis of data presented in the literature. In 2015, the groups of Elrod JW and Molkentin JD, independently showed how the inducible and conditional loss of cardiac MCU in a mouse model of I/R limits mitochondrial Ca^2+^ overload, the irreversible opening of mPTP and thus cell death; as consequence, the hearts resulted to be protected from bigger IS in reperfusion [24, 49]. By contrary, by taking advantage from a mouse model carrying a total deletion of MCU, Finkel’s group revealed the absence of IRI prevention. Hearts from WT and MCU^−/−^ mice had the same levels of apoptosis, cell contracture and no differences in mPTP opening [50]. However, although MCU depletion limited mitochondrial Ca^2+^ uptake also in this animal model, the basal metabolism remained unchanged. It cannot be hidden that data reported until now are controversial. Different results might be explained by the mouse model studied: whether MCU is deleted in the whole body, the onset of some compensation mechanisms might interfere with the physio pathological readout; currently, the inducible and conditional loss of cardiac MCU can be the most reliable method to assess mitochondrial Ca^2+ ^role during ischemia.

A very recent work by Ashok et al., demonstrated that mitochondrial NCX is the primary way of Ca^2+ ^entry in mitochondria of neonatal mouse ventricular myocyte knock-out for MCU [51]. This work supports the hypothesis of the adaptation of the mouse model carrying a germline deletion of MCU, in which Ca^2+ ^entry in mitochondria might be controlled in different ways.

More recently, without the use of transgenic preclinical models Guan L and colleagues reported a significant upregulation of MCU in IRI, confirming that the resulting mitochondrial Ca^2+^ overload led to dysregulation at multiple levels including the imbalance of mitochondrial quality control mechanisms like fusion, fission and mitophagy and the activation of calpains [52].

MCU is not the only one pore-forming protein of the complex. Also, MCUb plays crucial roles in Ca^2+^ channeling and it is considered as the dominant negative subunit of MCU [53]. To confirm in alternative ways the role of mitochondrial Ca^2+^ in I/R, in 2020 Molkentin JD and colleagues replicated the study carried out in 2015 to understand the behavior of this subunit under I/R conditions [54]. He described a significant upregulation of MCUb starting from 3 days after ischemia without detecting the protein at resting state or early at reperfusion time [54]. Although this could mean that MCUb adaptations can have protective roles during later stages of reperfusion, the exact ratio among MCU – MCUb – EMRE and its significance should be carefully evaluated under these experimental conditions.

### Ca^2+^ dysregulation at MAMs in myocardial infarction

MAMs are specialized regions where mitochondria and the SR come close together. Recent advancements in biomedical techniques have allowed a better visualization of this compartment in living cells using fluorescence confocal microscopy. MAMs act as biochemically independent areas while serving as communication points between SR and mitochondria, maximizing their signaling interactions. As we previously highlighted the importance of SR and mitochondria in AMI, it’s worth noting that MAMs also play a crucial role in this context. Indeed, they constitute several microdomains with high Ca^2+^ concentrations, thus being essential for cardiomyocytes in regulating Ca^2+^ transfer to mitochondria and supporting energy production. Thanks to the use of electron tomography, it has been demonstrated that SR and mitochondria are connected by tethers with a distance of approximately 10–30 nm and it has been defined a fundamental reliance of cell function and viability on the preservation of appropriate spacing between the SR and mitochondria [55].

Evidence suggests that dysfunctions at MAMs may contribute to the pathogenesis of reperfusion injury following AMI, suggesting that a decrease in the tethering between the two organelles is associated with a disturbance of clearance and ER stress, and a decline of mitochondrial dynamics included mitochondrial Ca^2+^ uptake, which, in turn, contributes to a decrease in cell death in cardiac myocytes subjected to I/R [56].

From a historical point of view, the first protein complex identified at MAMs was the IP3Rs/GRP75/VDAC1 axis. The different isoforms of the ER membrane IP3R are linked to voltage-dependent anion channel 1 (VDAC1) localized at OMM by glucose-regulated protein 75 chaperone (GRP75). This complex plays a critical role in enabling Ca^2+^ transfer between the two organelles, consequently controlling either the apoptotic process or the energy production. Specifically, IP3R3-GRP75-VDAC1 complex is implicated in mitochondrial Ca^2+^ overload and subsequent cardiomyocyte death observed during the reperfusion phase after sustained ischemic insult. Furthermore, the mitochondrial matrix protein Cyclophilin D (CypD) is also known as a regulator of mPTP activity [46], can interact with IP3R1-GRP75-VDAC1 complex. This interaction acts to regulate Ca^2+^ exchange from SR to mitochondria (Fig. 1) [57]. Proofs highlighting the relevance of VDAC1 in this pathological context come from a study carried out on a rat model of MI and on human cardiac tissues obtained from post-MI patients. The findings from these investigations unveiled the upregulation of VDAC1 levels; when inhibited through the oligomerization inhibitor VBIT-4 it relieves the increased fibrosis in the atrial myocardium of rats subjected to MI [58]. Further investigations have focused on glycogen synthase kinase-3β (GSK3β), which in its active form can phosphorylate VDAC1 and increase mitochondrial Ca^2+^ uptake. Moreover it has been revealed that in perfused rat hearts treated with GSK inhibitors, the ischemic process is less harmful on heart function [59, 60].

FUN14 domain containing 1 (FUNDC1) is a highly conserved OMM protein which plays a crucial role at MAMs: it is activated under ischemic conditions to induce mitophagy; while after reperfusion it is phosphorylated and inactivated resulting in reduction of mitophagy and activation of apoptosis [61]. FUNDC1 interacts with IP3R2 to control ER Ca^2+^ release into mitochondria; indeed, when depleted it leads to disruption of MAMs and to mitochondrial dysfunction [62]. Cardiomyocyte-conditional deletion of FUNDC1 provokes heart failure in vivo, which is worsened by acute MI: at molecular level this is explained by the disruption of MAMs integrity, showed as a decrease of MAMs proteins expression and a dissociation between mitochondria and ER, and a consequent impairment in Ca^2+^ homeostasis [62]. Other evidence both in vitro and in vivo confirmed the significance of FUNDC1 in myocardial injury following ischemic events, its expression and the mitophagic control are repressed leading to mitochondrial impairments and cardiomyocyte apoptotic death [63].

A key protein is also represented by Mitofusin 2 (MFN2), usually involved in controlling mitochondrial fusion, it is localized at ER and OMM interface and through the formation of homo- or hetero-dimers with MFN1 or MFN2 promotes MAMs stabilization (Fig. 1). Mitofusins revealed to be essential in cardiovascular system as they control mitochondrial fusion and ensure the correct mitochondrial morphology necessary for cardiac respiration and contraction [64].

MFN2 exhibits contrasting roles in MI and myocardial IRI. Some evidence suggests that MFN1/MFN2 double-knockout mice died at the embryonic stage due to lethal cardiac damage and conditional deletion in adult mouse hearts leads to severe impairments in mitochondrial functionality and cardiac respiration. Despite that, hearts deficient in both MFN1 and MFN2 are protected against acute IRI, due to an increased resistance to mPTP opening in response to Ca^2+^ and a reduction in mitochondria–SR interaction, suggesting that hearts deleted of both proteins are resistant to AMI [65]. Notably, MFN2-deficient hearts showed a less severe phenotype compared to double MFNs knockout, suggesting a potential compensation by MFN1 in mitigating the loss of MFN2. It has been shown that the deletion of MFN2 protected mice hearts delaying mPTP opening after Ca^2+^ overload and the following cell death after IRI [66]. MFN2 strongly impacts also on platelet biology as it normally preserves mitochondrial and complex I activities [67]. Its absence determines reduced respiration and ROS generation, all determinants involved in oxygen consumption needed for thrombin-dependent procoagulant functions and mPTP opening, thus decreasing platelet response during I/R and limiting IS [67].

A very recent work by Yepuri et al. showed that DIAPH1, a Formin involved in generation of actin filaments, directly interacts with MFN2 and regulates mitochondria–SR tethering. Once defined the localization of DIAPH1 in mitochondria and at SR, it has been shown that its silencing leads to disruption of the tethering between these two organelles and the consequence is a protection from I/R both in cardiomyocytes and beating hearts [68]. This modulation of myocardial IRI has been previously demonstrated showing that DIAPH1 deletion regulates serum response factor (SRF) and early growth response 1 (EGR1) leading to modulation of Ca^2+^ transporters in cardiomyocytes such as SERCA2a expression [69].

However, there are also some controversies in the scientific literature about MFN2 knockout models. A cardiac MFN2 knock-out mouse exhibited a worsened response to IRI, due to an impairment of mitochondrial functionality and cellular homeostasis; in fact, the lack of this protein leads to increased accumulation of autophagosomes in response to I/R stress and to cardiac dysfunction [70]. Further research is needed to elucidate the role of MFN2 in the protection against IRI after MI, especially because recent discoveries bring to light different MFN2 variants essential to shape the ER or to tether it to mitochondria and it has to be clarified which of them is involved in pathobiology of IRI [71].

Another essential complex involved in proper functioning of MAMs is B cell receptor–associated protein 31 (BAP31)-Mitochondrial fission 1 protein (Fis1). Fis1 is an OMM protein, creating a complex involved in an early event during apoptosis induction, it transmits an apoptotic signal from the mitochondria to the ER by interacting with BAP31 and leading to its cleavage into the pro-apoptotic form p20BAP31 after the recruitment of procaspase-8. This apoptotic message triggers the release of Ca^2+^ from the ER to the mitochondria for apoptotic cascade activation [72]. During I/R, p20BAP31 form causes Bax and Bak translocation to the OMM and Cyt-C release from the mitochondria, these events lead to cardiomyocytes death and provide further evidence of the involvement of Ca^2+^ handling at MAMs in the pathophysiology of MI [73].

The regulation of MAM structures involves the tethering between SR-resident protein Vesicle-associated membrane protein-associated protein B (VAPB) and mitochondrial protein tyrosine phosphatase-interacting protein-51 (PTPIP51) (Fig. 1) [74]. This interaction is fundamental for the control of Ca^2+^ homeostasis and autophagic process [75].

The Sigma-1 receptor (Sig-1R) is a transmembrane protein of the SR, it mostly resides at MAMs where it interacts with binding immunoglobulin protein (BIP); in cases of Ca^2+^ depletion from the SR, Sig-1R dissociates from BIP and binds to IP3Rs leading to a prolonged Ca^2+^ influx into mitochondria [76].

In conclusion, the study of MAMs in AMI sheds light on the complex interplay between the SR and mitochondria and that Ca^2+^ takes essential roles in cardiac contraction and cell fate in response to ischemic events. Further studies into the mechanisms underlying MAMs function and dysfunction might potentially pave the way for the development of targeted interventions for future AMI therapies. Of note, the experimental work by Csordas G and colleagues, consisted in generate an engineered mitochondria-SR tether (linker) in mice which induced a strong mitochondrial and SR remodeling, enhancing the contacts, but also showed an increase resistance to injury in vivo and ex vivo I/R [77]. A specific transgene for cardiac muscle was introduced into mice, containing a monitorable protein. Through in vivo and in vitro clinical tests, the behavior of the transgene was identified, showing both expanding and contracting actions on mitochondrial-SR contacts, reducing mortality of cardiac muscle cells and damage from Ca^2+^ overload in I/R situations. This process balances the interaction between SR and mitochondria, enhancing resilience to stress conditions associated with abnormal Ca^2+^ regulation [77].

### Cardioprotective strategies targeting Ca^2+^ signaling at MAMs

Over the years, a wide range of experimental strategies have been employed to improve patient outcomes in AMI clinics following IRI [78, 79]. The existing therapies are mainly aimed to prevent cardiac overload and fatigue after the ischemic episode; they also work to reduce the occurrence of secondary events by managing main risk factors, such as hypertension and atherosclerosis. However, many molecular mechanisms becoming impaired during I/R damage, often linked to Ca^2+^ homeostasis and mPTP opening are currently far from being considered as much as they should be (Table 1). Indeed, while our understanding of basic and preclinical research is expanding, translational studies directed at signaling between the SR and mitochondria are in the growing phase and require further attention. The current clinical picture poses also further challenges, as many cardiologists believe that the decline in mortality has stabilized and remains uncertain whether it can be reduced in either the short or the long term. Considering these issues, is it possible to study SR and mitochondrial environment and their contact sites as a therapeutic target in the pathophysiology of AMI?

**Table 1:** Modulators of the Ca^2+^ signaling with cardioprotective effects in AMI

Modulators	Protein target	Molecular effects	Physiologic effects
RuRed [81]	MCU	Inhibition of mitochondrial Ca^2+ ^overload	Improved cardiac contractility
Ru360 and Ru265 [82]			Reduced RI, prevention of arrhythmias and mitochondrial damage, increased heart contractility
KB-R7943 [85]	MCU/NCX	Inhibition of mitochondrial Ca^2+^ load, inhibition of Ca^2+^-induced mPTP opening, inhibition of the reverse mode of NCX	Protective effects following I/R by improving LV function, by reducing ventricular fibrillation and hypercontracture of cardiomyocytes
DS16570511 [86]	MCU	Inhibition of mitochondrial Ca^2+ ^overload	Beneficial effects on increasing cardiac contractility and reducing IRI
MCU-i4 [87]	MICU1	Inhibition of mitochondrial Ca^2+ ^overload	
Melatonin [90, 91]	IP3Rs SERCA2a	Reduction of Ca^2+^ overload and inhibition of apoptosis	Improves the organization of actin filaments in cardiomyocytes against H/R
AVV/PTPIP51 [100]	PTPIP51	Reduction of mitochondria-SR contacts and Ca^2+^ exchange	Protected cardiac tissue after I/R
Resveratrol [103]	VDAC1	Increased SERCA2a activity and Ca^2+ ^transients	Significantly improved LV pressure and coronary flow

#### Pharmacological regulation of mitochondrial Ca^2+^ intake

**Fig. 2:**
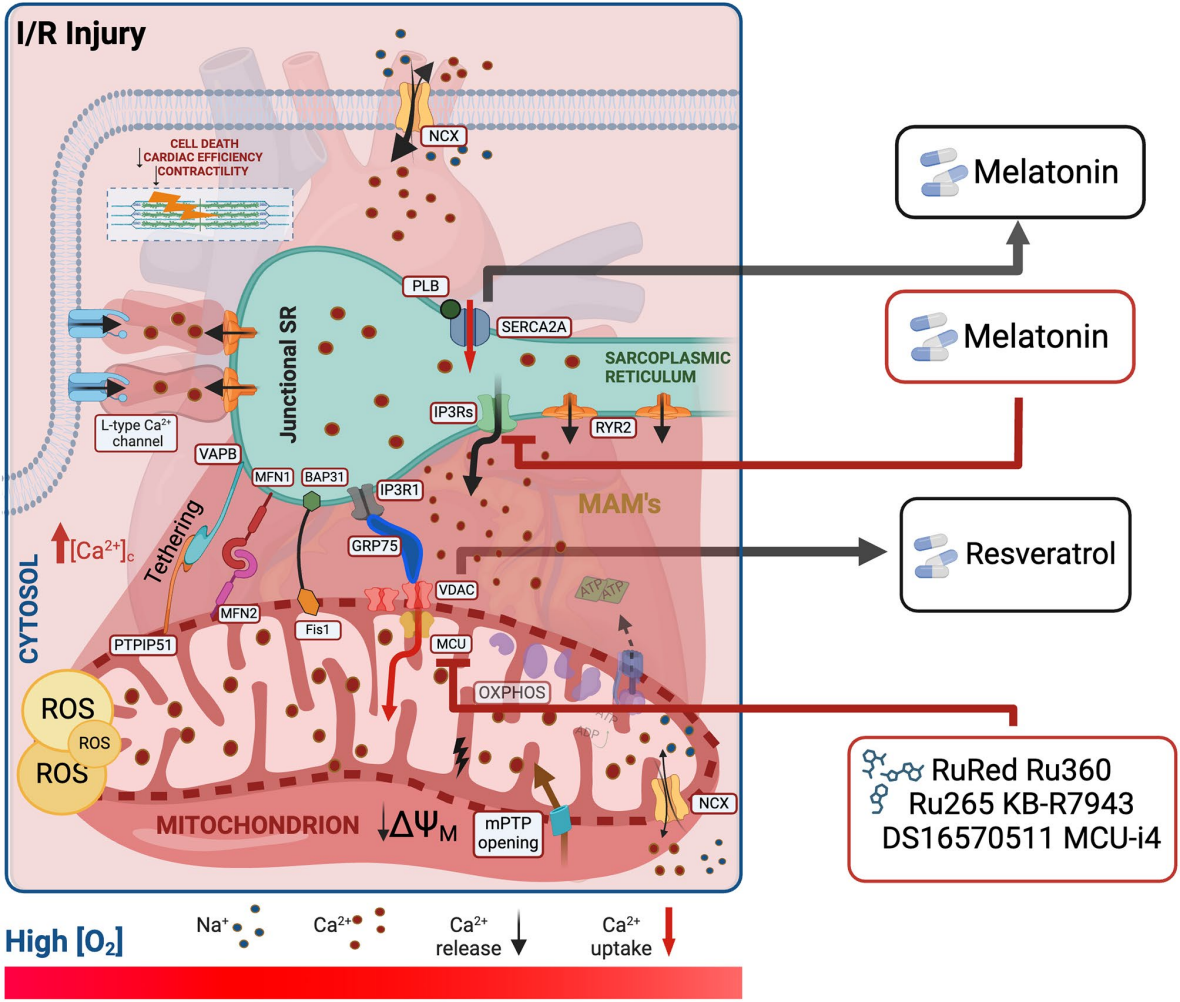
Representation of proteins involved in Ca^2+ ^signaling and possible related cardioprotective strategies to prevent ischemia-reperfusion damage This figure depicts the key proteins involved in calcium (Ca^2+^) signaling that are dysregulated during I/R and their potential cardioprotective strategies to mitigate I/R demange Sarcoplasmic Reticulum (SR), Mitochondria associated membranes (MAM), Mitochondrial calcium uniporter (MCU), Voltage-dependent anion-selective channel (VDAC), Inositol 1,4,5-triphosphate receptor type 2 (IP3R2), Inositol 1,4,5-triphosphate receptor type 1 (IP3R1), Ryanodine receptors (RyR2), Plasma membrane Ca^2+ ^ATPase, sarcoplasmic reticulum Ca^2+ ^ATPases (SERCA), Phospholamban (PLB), Glucose-regulated protein 75 chaperone (GRP75), Mitofusins 1 and 2 (MFN1 and MFN2), Vesicle-associated membrane protein-associated protein B (VAPB), Protein tyrosine phosphatase-interacting protein-51 (PTPIP51), B cell receptor–associated protein 31 (BAP31), Mitochondrial fission 1 protein (Fis1)

After its discovery, MCU targeting appeared the best reliable way for a direct therapeutic approach to treat the excessive Ca^2+^ intake into the mitochondrion during I/R. Several groups have developed molecules capable of reducing mitochondrial Ca^2+^ uptake, the first one was ruthenium red (RuRed), synthesized in 1892. RuRed is a compound that inhibits MCU without affecting Ca^2+^ efflux and mitochondrial respiration [80, 81]. The most important study investigating the use of RuRed in heart disease was by Grover et al. in which the treatment of rat hearts perfused with the compound in the micromolar range improved cardiac contractile function and oxygen reperfusion efficiency after ischemia [82]. Due to its low selectivity and permeability, RuRed has come under criticism over the years and stimulated the development of some additional derivatives like Ru360 and Ru265 with improved properties, like higher selectivity, cell permeability accompanied by low toxicity [83] (Table 1). MCU inhibition has been reached also by using KB-R7943, a compound originally developed as an inhibitor of the NCX [84], that has been shown to protect against myocardial IRI through the inhibition of MCU causing a reduced Ca^2+^ uptake within the mitochondrial matrix (Fig. 2) [84]. Studies carried out on rats have demonstrated that using KB-R7943 at concentration of 1µM had a protective effect in IRI through restoring left ventricular (LV) function, by reducing ventricular fibrillation and hypercontracture of cardiomyocytes [85].

DS16570511 and MCU-i4 synthesis recently updated the list of mitochondrial Ca^2+^ uptake inhibitors, giving to the target an appealing route and a good pharmacological profile to be translated in clinic [86, 87]. The use of DS1657051 in the 3–30 µM range blocked mitochondrial Ca^2+^ uptake with beneficial effects on increasing cardiac contractility and reducing IRI. An interesting property is reversible binding with the target after washout [86]. On the other hand, MCU-i4 binds a specific domain of MICU1, fundamental for the gating activity of MCU complex, but it affects mitochondrial depolarization (Fig. 2). Mainly at preclinical level, these treatments led to rapid beneficial effects like increased heart contractility and reduced IS [88] However, while these advancements are exciting, it’s crucial to approach them with a critical lens. Further research is necessary to fully elucidate their mechanisms of action, assess their safety profiles, and evaluate their efficacy in clinical trials. Their effects on mitochondrial depolarization and the necessity for translation to clinical settings warrant careful consideration.

#### Pharmacological regulation of SR Ca^2+^ intake and release

Chronic hypoxia leads to the deterioration of myocardial functions with impaired Ca^2+^ handling in charge of the SR, which may be mediated by oxidative stress. Studies have hypothesized that antioxidant administration would protect against cardiac IRI by improving SR Ca^2+^ management [89].

On the most known natural compounds and hormone acting at SR levels is Melatonin; the authors of this study [90] investigated its effects on intracellular Ca^2+^ handling as therapeutic strategy to counteract acute cardiac injury-related side effects. Taking advantage from the establishment of an in vivo mouse model of IRI, they described how Melatonin can impact on both SR and mitochondria subcellular compartments. Indeed, it is able to inhibit the IP3R phosphorylation and, at the same time also MCU expression, thus alleviating both cytoplasmic and mitochondrial Ca^2+^ overload which occurs after ischemia (Fig. 2). Melatonin appears to have additional properties without showing a peculiar molecular pathway; it has been shown to downregulate IP3R expression and to promote SERCA2a expression via the ERK1 pathway in cardiomyocytes preventing I/R damage. However, the lack of elucidation on its specific molecular pathway raises questions about its precise mode of action. Melatonin also inhibits apoptosis of cardiomyocytes and improves the organization of actin filaments in cardiomyocytes subjected to I/R [91]. As mentioned above, SERCA2a plays an important role in bringing Ca^2+^ from cytosol to SR lumen. Reduced SERCA2a activity and expression is one of the consequences that occur following IRI. Using viral vectors to promote its long-term overexpression in cardiac tissue was observed to markedly enhance cardiac function and reduce myocardial apoptosis. This improvement was ascribed to the augmentation of Ca^2+^ signaling, directly impacting the functionality of vascular endothelial cells and smooth muscle [92]. Studies conducted on rats have demonstrated that lentiviruses can integrate the gene of interest into the host genome with a long-term effect of genetic transduction [93]. Even in I/R models, overexpression of SERCA2a reduced the incidence of ventricular arrhythmia and improved hemodynamics [94, 95].

A recent study conducted in the field of cardiac function, showed that phosphorylation at serine 663 of SERCA2 emerges as a pivotal clinical and pathophysiological occurrence. Elevated phosphorylation at this site is evident in ischemic hearts of both patients and preclinical models. Inhibiting serine 663 phosphorylation, through a CRISPR/Cas9-mediated genome editing strategy allowed to generate a human cell line expressing a phosphoresistant SERCA2 mutant (SERCA2^S663A^), enhances SERCA2 activity, offering protection against cell death by mitigating cytosolic and mitochondrial Ca^2+^ overload [96]. The above-mentioned necrosis and apoptosis are only two of the plethora of cell death mechanisms by which cells die after stressors [97] and in which Ca^2+ ^and inflammation are crucial factors. Parthanatos is one type of programmed necrotic cell death mainly described in neurodegenerative diseases and stroke and appropriately reviewed in [98]. Important molecular mechanisms behind Parthanatos relies on excessive intracellular ROS accumulation and an abnormal and dependent ER Ca^2+^ release directed to mitochondria; as consequence, mitochondrial membrane depolarizes and an irreversible energetic crisis occurs [99]. The authors did not investigate the proteins involved in this wrong ER-mitochondria Ca^2+ ^communication, but found in Propofol a potent pharmacological agent able to counteract ROS production and ER-mediated Ca^2+ ^release with beneficial outcomes against Parthanatos-mediated IRI [99].

From the literature, Propofol results to exert a protective action also in cardiac-related settings (i.e., open-heart surgery, cardioplegia, valve replacement, I/R). Almost all studies ascribe the effect to the increase in the antioxidant defense of the organism; no mention to specific Ca^2+ ^signaling pathways being involved. It would be interesting to expand this knowledge in disease, to confirm the ER-mitochondria modulation of Ca^2+ ^transfer and to unveil the responsible proteins.

#### Direct regulation of MAMs

Despite their importance, still few drugs have been reported to modulate Ca^2+^ signaling and architecture within MAMs. To the best of our knowledge, it has only been studied the involvement of PTPIP51 in regulating cardiac function controlling the mitochondria-SR junction. Studies performed on I/R mouse hearts, showed that the adenovirus-associated virus (AAV)-mediated knockdown of PTPIP51 significantly protected cardiac tissue after I/R due to a reduction of mitochondria-SR contacts and Ca^2+^ exchange [100] (Table 1).

The fact that only PTPIP51 has been studied overlooks potential alternative targets within MAMs, limiting our understanding of their modulation. Additionally, the reliance on a single study to support PTPIP51’s efficacy in I/R injury may present an oversimplified view of MAM involvement in cardiac function. Further exploration of other molecular targets within MAMs is warranted to fully understand their role in cardiac pathology and to develop more effective therapeutic strategies.

Recently, some reports are describing a change in expression of IP3Rs receptors in various cardiac diseases, as a maladaptive response in charge of the MAMs [101]. Indeed, in vivo knockdown of IP3R in murine hearts protected against MI and induced cardiac structural remodeling and fibrosis [102]. Studies conducted on hearts in which global ischemia is generated by the Langendorff system, have shown that the drug Resveratrol significantly improved LV pressure and coronary flow. The activity of lactate dehydrogenase and creatine phosphokinase were also decreased resulting in a reduction in IS. Resveratrol is also known to inhibit the upregulation of the expression of the anion channel VDAC1 triggered by IRI, confirming that VDAC1 plays an important role in resveratrol-mediated cardioprotection [103]. CypD seems to have a double role, being a modulator of the mPTP and triggering its opening following an increased accumulation of Ca^2+^ in the mitochondrial matrix [104]. Moreover, during I/R, CypD would synergistically act with the IP3R1-GRP75-VDAC1 complex and with an increase in mitochondrial Ca^2+^ load and induction of apoptosis. Inhibition or downregulation of CypD, prevented mitochondrial Ca^2+^ overload and cell death in an adult mouse cardiomyocyte I/R model [57]. In this context it should be reported also that CypD negatively regulates mitochondrial Ca^2+^ accumulation [105]. So, in contrast to what wrote above, CypD would actively participate in a pathological increase of mitochondrial Ca^2+^ which determines glucose rather than fatty acids oxidation with alterations in cellular bioenergetics and mPTP activity [105].

In cardioprotection mechanical strategies exist besides pharmacological treatments [106]. This is the case of the ischemic preconditioning (IPC) and the ischemic postconditioning (IPostC) in which brief episodes of intermittent I/R before or after the main ischemic event, respectively, protect the heart from the main features of reperfusion damage [106]. Although direct evidence about the impact of IPC and IPostC on SR-mitochondria tethering do not exist yet, it is known that these techniques concur to minimize ATP depletion, ROS generation and thus mitochondria-related dysregulations. Multiple studies involving the use of cultured cardiomyocytes and preclinical models of cardiac I/R confirmed that repeated interruption of blood reflow at reperfusion time (i.e., 3 cycles of 5 min hypoxia/reoxygenation) decreases mainly oxidative stress but also both cytosolic and mitochondrial Ca^2+^ overload [107] with the preservation of mitochondrial membrane potential [108] and a strong inhibition of mPTP opening [109]. The prevention of mitochondrial Ca^2+^ overload probably refers to the blockade of excessive Ca^2+^ release from SR, as investigated by Khalaf A. and co-workers by using a similar protocol of IPostC [110]. Instead, the increase in intracellular Ca^2+^ persistent can be avoided by the IPostC-mediated increase in PLB phosphorylation which guarantees a correct SR Ca^2+^ load [111]. To sum up, there is a deficiency in effective therapeutic approaches specifically targeting Ca^2+ ^transfer at MAMs in myocardial reperfusion injury. However, among the strategies identified as protective in patients, most of their beneficial effects can be directly or indirectly attributed to the modulation of Ca^2+^-mediated damage. While there is growing interest in targeting MAMs to modulate Ca^2+ ^signaling, the limited knowledge underlines the needs of further investigation to optimize the therapeutic potential both in preclinical and clinical settings.

## Conclusions

The research on SR and mitochondria, and on their contacts, has highlighted the important role of these organelles in the pathophysiology of CVDs, especially in AMI. Although the molecular characterization of these pathways has reached the golden age, we must admit that the road to fully understand how they can be successfully targeted in therapy is still far. What we know is that the disruption of the architecture of these subcellular sites occurs during AMI and can lead to further damage to the heart muscle in preclinical models. Likewise, the use of Ca^2+^ handling/cycling correctors can help to protect the organelles from damage and to restore the normal function of the cardiac tissue. Despite that, we cannot understand the real impact on the clinical outcome of the patients affected, whether the translational studies and clinical trials do not give feedback to the scientific community.

### Electronic supplementary material

Below is the link to the electronic supplementary material.

Supplementary Material 1

Supplementary Material 2

Supplementary Material 3
