# Supplementary material for: Calcium signaling from sarcoplasmic reticulum and mitochondria contact sites in acute myocardial infarction
Source: J Transl Med. 2024 Jun 9;22:552. doi: 10.1186/s12967-024-05240-5 (PMC11162575; doi:10.1186/s12967-024-05240-5)
Supplement: Supplementary file 1 — Supplementary Material 1 [file 12967_2024_5240_MOESM1_ESM.docx]

**Keywords**: acute myocardial infarction, mitochondria associated membranes, mitochondria, sarcoplasmic reticulum , ischemia reperfusion Injury

**Abbreviations**

AAV Adenovirus-associated virus

AMI Acute myocardial infarction

BAP31 B cell receptor–associated protein 31

BIP Binding immunoglobulin protein

BKCa Large-conductance calcium-activated potassium ion channels

Ca^2+^ Calcium ion

CaMKII Ca^2+^/calmodulin-dependent protein kinase II

CVD Cardiovascular diseases

CypD Cyclophilin D

ECC Excitation-contraction coupling

ETC Electron transport chain

Fis1 Mitochondrial fission 1 protein

FUNDC1 FUN14 domain containing 1

Grp75 Glucose regulated protein 75

GSK3β Glycogen synthase kinase 3β

GTP Guanosine triphosphate

H/R Hypoxia-reoxygenation

HF Heart failure

IMM Inner mitochondrial membrane

IP3R Inositol 1,4,5-trisphosphate receptor

IPC Ischemic preconditioning

IPostC Ischemic postconditioning

IRI Ischemia reperfusion Injury

IS Infarct size

IDH Isocitrate dehydrogenase

IHD Ischemic heart diseases

JPH Junctophilins

KGDH Ketoglutarate dehydrogenase

LV Left ventricular function

MAMs Mitochondria associated membranes

MCU Mitochondrial calcium uniporter complex

Mfn1 Mitochondrial fusion proteins 1

Mfn2 Mitochondrial fusion proteins 2

MI Myocardial infarction

mPTP Mitochondrial permeability transition pore

mtROS Mitochondrial ROS

MPT Mitochondrial permeability transition

NCX Na^+^/ Ca^2+^ antiporter

OMM Outer mitochondrial membrane

PTPIP51 Protein tyrosine phosphatase interacting protein 51

PCI Percutaneous coronary intervention

PDH Pyruvate dehydrogenase

PKC Protein kinase C

PLB Phospholamban

PTPIP51 Mitochondrial protein tyrosine phosphatase-interacting protein-51

ROS Reactive oxygen species

RyR2 Ryanodine receptor 2

SERCA2a Sarco-Endoplasmic Reticulum Calcium ATPase

Sig-1R The Sigma-1 receptor

SR Sarcoplasmic reticulum

SRF Serum response factor

STEMI ST-segment elevation myocardial infarction

TCA Tricarboxylic acid

VAPB Vesicle-associated membrane protein-associated protein B

VDAC Voltage-dependent anion-selective channel
