# Supplementary material for: Calcium signaling from sarcoplasmic reticulum and mitochondria contact sites in acute myocardial infarction
Source: J Transl Med. 2024 Jun 9;22:552. doi: 10.1186/s12967-024-05240-5 (PMC11162575; doi:10.1186/s12967-024-05240-5)
Supplement: Supplementary file 3 — Supplementary Material 3 [file 12967_2024_5240_MOESM3_ESM.pdf]

Università degli Studi di Ferrara
Dipartimento di Scienze Mediche

Università degli Studi di Ferrara
Dipartimento di Scienze Mediche
Via Luigi Borsari, 46 - 44121 Ferrara
Telefono: 0532 – 293321 email: rsolsn@unife.it
pec: dipscienzemediche@pec.unife.it
Partita Iva 00434690384 - Codice Fiscale 80007370382

Ferrara, April 9th 2024

Dear Editor-in-Chief Prof. Francesco Marincola,

I would like to submit a revised version of our review manuscript entitled ***"Calcium signaling from sarcoplasmic reticulum and mitochondria contact sites in acute myocardial infarction"*** by Agyapong et al. for your kind consideration and for publication in Journal of Translational Medicine.

We carefully checked all reviewers' concerns and we provided all appropriate modifications as requested. Please, see also the "Response to reviewers" file. Overall, we totally agree with the questions made by the reviewers and we truly thank them. Now the manuscript appears more focused and complete.

This manuscript has not been submitted elsewhere and all authors accepted this submission.

Kind regards,
Giampaolo Morciano, PhD, FESC
