# Supplementary material for: Calcium signaling from sarcoplasmic reticulum and mitochondria contact sites in acute myocardial infarction
Source: J Transl Med. 2024 Jun 9;22:552. doi: 10.1186/s12967-024-05240-5 (PMC11162575; doi:10.1186/s12967-024-05240-5)
Supplement: Supplementary file 2 — Supplementary Material 2 [file 12967_2024_5240_MOESM2_ESM.docx]

Reviewer #1: Overall, the topic of this review is of relevance for the scientific community and I think worth being published. However, the following minor concerns should be addressed

The following pertinent reports should be mentioned/discussed:
doi: 10.1161/CIRCRESAHA.123.322914
doi: 10.1016/j.yjmcc.2022.11.003.
doi: 10.1073/PNAS.1513047112.
doi: 10.1161/CIRCRESAHA.119.316306.
doi: 10.1038/s41419-018-0996-9.
doi: 10.1007/978-3-319-55330-6_10.
doi: 10.3390/antiox9080760.
doi: 10.1093/cvr/cvs116.

*We thank the reviewer for his/her consideration about our manuscript and we appreciated your attention to details; accordingly to your suggestions, we added the references requested along the manuscript. We hope that the important information provided may attract more interest from the reviewer and the readers. All changes have been highlighted in red in the new version of the text.*

The presentation and critical interpretation of results of previous studies should be improved.

Enhancements are needed in presenting and critically interpreting the results of previous studies.

*We thank the reviewer for his/her comments. We took advantage from these suggestions to improve the main text by adding further interpretations to the facts, especially in the last chapter that aims to discuss the cardioprotective strategies related to the SR-mitochondria interface.*

Additionally, the quality of the figures should be enhanced, incorporating informative elements for Readers

*We thank the reviewer for his/her comment. Accordingly, we improved the figures by adding the figures legend and highlighting and better explaining the differences between what occurs in healthy and IR injury conditions.*

Reviewer #2: Authors performed an accurate and extensive analysis with the aim to summarize the knowledge about MAMS regulation in AMI, focusing in particular on the Ca2+ signaling. Authors further offer an overview about possible cardioprotective targets to limit the deregulation of the Ca2+ signaling in AMI. The manuscript is well structured and complete and sincerely highlights the controversial data present in literature.

*We truly thank the reviewer for this comment, we appreciated this kind consideration. All changes have been highlighted in red in the new version of the text.*

My suggestions for Authors is only to discuss some points more broadly, to implement the completeness of the text and the understanding of people not experts in the topic:

- page 3: Describe what mitochondrial enzymes are influenced by the concentration of Ca2+ and how their regulation promotes electron transport and ATP production.

*We thank the reviewer for the valuable comment. The reviewer is right. Accordingly, we added information about Ca^2+^-dependent enzymes in supporting mitochondrial activity and ATP production.*

- page 3: Better explain what type of mechanisms of mitochondrial protection are activated by RyR2 and how the increase in Ca2+-dependent mitochondrial ROS can lead to RyR2 oxidation.

*We thank the reviewer for the comment; we gave better description on how RyR2 is involved in active mitochondrial protection.*

- page 4: Giving the results reported in the manuscript for the MCU-/- mice, in contrast with the data on the inducible and conditional loss of cardiac MCU, Authors think about a compensatory effect occurring in MCU-/- mice?

*Yes, exactly. In the new version of the text we further discussed this issue. We thank the reviewer for the comment.*
